# Motivating Factors and Potential Deterrents to Blood Donation in High School Aged Blood Donors

**DOI:** 10.1155/2016/8624230

**Published:** 2016-05-15

**Authors:** Rachel Finck, Alyssa Ziman, Matthew Hoffman, Michelle Phan-Tang, Shan Yuan

**Affiliations:** Department of Pathology and Laboratory Medicine, David Geffen School of Medicine, University of California, Los Angeles, Los Angeles, CA 90095, USA

## Abstract

*Background*. To ensure an adequate supply of blood, collection centers must design campaigns that successfully recruit and maintain an active donor pool. Understanding factors that motivate and deter individuals from donating may help centers develop targeted recruitment campaigns. These factors among high school aged blood donors have not yet been fully investigated.* Study Design and Methods*. A voluntary, anonymous survey was administered to student donors at high school mobile blood drives. The survey instrument asked the students to rate several potential motivating factors in their importance in the decision to donate blood and several potential deterring factors in their future decision whether or not to donate blood again. The survey also asked the students to rate the desirability of several potential incentives.* Results*. Motivating factors that reflected prosocial, empathetic, and altruistic thoughts and beliefs were rated highly by students. Pain from phlebotomy was most commonly chosen as potential deterrent. Movie tickets and cookies/snacks at the drive were rated as the most attractive incentives.* Conclusion*. High school aged blood donors are similar to other donor groups in their expressed motives for donating blood. This group may be unique in the factors that deter them from donating and in their preferences for different incentives.

## 1. Introduction

In recent years, the incidence of red blood cell (RBC) transfusions in the United States has declined; a 2013 survey by the AABB estimated that the number of annual whole blood/RBC transfusions was 6.1 million units, representing a 7.3% decrease compared to 2011. This survey also found that multidisciplinary patient blood management (PBM) systems are increasingly common; among US hospital respondents, 547 responded positively to having implemented PBM systems, compared to 529 in 2011 [1]. Among other strategies, PBM includes establishing protocols that promote the use of evidence-based hemoglobin thresholds in the decision to transfuse blood [2]. As mounting evidence suggests that restrictive transfusion strategies are noninferior to liberal strategies for some groups of patients [3–6], these systems encourage more judicious use of blood by avoiding unnecessary transfusions and complications associated with them. In tandem, with fewer transfusions, blood donations have also decreased. Collections in the US reached a peak in 2008 but have been declining ever since. In 2013, total allogeneic whole blood collections in the US were significantly lower (12.8% decrease) than they had been in 2011 [1].

Nonetheless, the demographic composition of the United States population is changing. Census data predicts that individuals aged 65 years and older will comprise an increasingly large percentage of our population, as are persons of a racial or ethnic minority [7]. As the population ages, blood transfusions may also increase, as a disproportionately larger percentage of the population will be at higher risk for malignancies, chronic illnesses, and other clinical states requiring surgical intervention, all of which are associated with an increased need for blood transfusion [8]. However, as blood donation has diminished in recent years, if future collection efforts are not augmented to supply this potential demand for blood, blood shortages could occur. An additional challenge exists in the fact the RBC antigen profile varies among ethnicities; this impacts the effort to provide transfusion support to alloimmunized patients of different ethnicities, especially those who may need chronic transfusions [9]. In order to maintain a blood supply over a period for an ethnically diverse population, collection facilities must appeal to a donor pool of similar diversity. Thus, US blood collection facilities may face the challenge of expanding their collection programs to maintain the supply for a diversifying demand.

In this country, blood collection facilities rely on a pool of active, dedicated volunteer blood donors. To maintain a replenishing blood supply, it is important to both induct first-time donors and enable repeat donors to continue their donation behavior. Recruitment strategies should be designed with these goals. Similar to advertising, recruitment campaigns may be most successful when they are developed with an understanding of the specific beliefs and desires of those they seek to recruit. Highlighting aspects of blood donation that are particularly motivating or attractive to specific demographic groups allows blood collection agencies to target their recruitment campaigns [10–12]. Additionally, borrowing from a customer relationship management concept of customer-focused service, blood collection facilities that venture to understand the thoughts and feelings of the individuals in their donor pool (or “customers”) may train their staff and nurses to interact with donors in ways that are most effective in engendering repeat donation behavior (or “repeat business”) [13].

Past studies have shown that significant differences exist between blood donors in the factors that motivate and deter them to engage in blood donation. Differences have been described between males and females, different races, age groups, levels of education, and extents of donation experience [10–12, 14–18]. Donors aged 16–18, however, remain an understudied population in the blood donation literature. For our blood collection program, high school blood drives are held throughout the academic school year, and whole blood units from these drives account for an average of 65% of our total collections during this time. Despite the abundance of this age group in our donor pool, relatively little is known about what factors impact their donation behavior.

This age group is particularly important to recruit as initial and repeat donors for several reasons. Their youth predisposes them to potentially long careers as blood donors. They are less likely to have many of the factors or activities that cause deferments in older donors, such as illnesses, travel, or high risk sexual activity. In Los Angeles, they are also an ethnically diverse group that mirrors the diversity of the patient population that our center seeks to support. This study sought to survey student donors at high school blood drives conducted by the UCLA Blood and Platelet Center (UCLA BPC) to investigate what motivated them to donate blood, what might deter them from donating again, and which incentives they preferred in appreciation for their donation.

## 2. Methods

The survey was written by the investigators and consisted of three sections (see the Appendix). First, it requested basic demographic information (sex, age, and race) and asked how many times the respondent had donated in the past. Then, respondents were asked to rate on a five-point scale the importance of various motivating and potentially deterring factors in their decision to donate blood. Finally, on a four-point scale, the respondents were asked to rate the appeal of various incentives offered by the blood collection facility.

The survey was administered in printed format at four blood drives conducted by the UCLA BPC at different high schools within the Los Angeles Unified School District (LAUSD) from January to May 2012. Participation was voluntary and restricted to students (parents and teachers who donated at the drive were not asked to participate). Investigators asked students who had just donated if they would like to fill out the survey during their mandatory 15 minute postdonation detainment at the canteen. Student donors that chose to participate were provided with an assent document that described the intent of the survey and the rights of the respondent. To incentivize participation, students that completed their surveys were entered into a raffle to receive a prize (a $50.00 gift certificate to a national chain electronics store) with one winner per school. To protect anonymity of the respondents, surveys were collected in a sealed box at the exit of the canteen. The sealed boxes were transported from the blood drives to the investigators' offices at the UCLA BPC for entry of responses into electronic format. The paper surveys were then destroyed. Statistical analysis followed.

This study, survey content, and assent document were approved by both the UCLA Institutional Review Board (IRB) and the Committee for External Research Review of the LAUSD. The UCLA IRB reviewed and waived the requirement to obtain parental consent or permission under 45 CFR 46.116(d) of the Federal Regulations.

### 2.1. Statistical Analysis

Each survey respondent was asked to rate the importance of motivating (question #(5)) and deterring factors (question #(7)) for blood donation, as well as the appeal of incentives to blood donation (question #(8)). Responses to each of these questions were dichotomized for the purpose of analysis. For questions #(5) and #(7), “very important”/“very influential” and “somewhat important”/“somewhat influential” were considered positive responses and “neutral/not sure,” “somewhat unimportant”/“not much influence,” and “very unimportant”/“no influence” were considered negative responses. For question #(8), “appealing” and “somewhat appealing” were added together as a positive response and “neutral” and “not appealing” were added as a negative response.

For each question, we computed the combined percentages of persons who answered positively and negatively by sex and race and overall. These percentages were compared by level of each factor using the chi-square test. For this analysis, each factor was examined independently, ignoring the other factor (i.e., percentages were compared across race, ignoring sex, and between sexes, ignoring race).

Following the initial analysis, we evaluated the simultaneous influence of sex and race on each of the binary outcomes to produce an adjusted analysis. To do so, we used the multiple logistic regression model that included sex and race as the main effects and sex × race as the interaction effect. The effect of each factor was therefore not presumed to be the same across the levels of the other factor. For question #(7), since some of the cell counts were zero and the logistic model is difficult to evaluate in the presence of zero cell counts, a corresponding nonparametric multiple regression model was used based on a bootstrapping procedure instead of the logistic model.

We computed the adjusted (marginal) proportions by sex and race under the above multiple logistic/regression models and the corresponding *p* values for (1) main effect of sex, (2) main effect of race, and (3) sex × race interaction effect. The *p* value was considered significant if less than 0.05. This adjusted analysis allowed us to evaluate each factor while controlling for the other factor and to evaluate whether there were any synergistic (nonadditive) effects between the two factors.

## 3. Results

Among four separate blood drives, we collected complete survey information from 395 blood donors; all were aged 16–19. Of these respondents, 206 (52%) selected female and 189 (48%) selected male. With regard to race distribution, 36 (9%) identified themselves as African-American, 76 (19%) as Asian-American, 71 (18%) as Caucasian, 186 (47%) as Hispanic/Latino, and 26 (7%) as mixed or other race. A total of 228 (58%) had not donated blood before, 75 (19%) had donated once before, 61 (15%) had donated 2-3 times before, and 31 (8%) had donated more than 3 times before (question #(4)).

Table 1 lists the adjusted percentages by sex and race and overall for questions #(5) (potential motivating factors for blood donation) and #(7) (potential deterrents for future blood donation) and the *p* values.

The most commonly chosen motivating factor, item (5)(a) “donating blood is the right thing to do” was rated as important by 90% of all respondents. The following additional items were rated as important by more than half of all the respondents in order of decreasing importance: item (5)(c) “shortage of blood” (72%), item (5)(b) “donating blood makes me feel like a hero” (65%), item (5)(i) “someone will be proud of me if I donate” (59%), and item (5)(e) “my friends are donating” (51%). Item (5)(j) “I want to help my school donate more than any other school” was rated positively by just less than half of all respondents overall (49.9%). Item (5)(l) “gift/incentive” was the least commonly chosen motivating factor (30%).

Items (5)(a) and (5)(e) were more likely to be rated as important by males than by females after adjusting for race. Item (5)(b) was more likely to be rated as important by African-Americans or Hispanics compared to other races after adjusting for sex. Items (5)(f) “others in my club/sport are donating,” (5)(g) “adults in my church/school/community donate,” and (5)(j) “I want my school to donate more than any other school” were most likely to be rated as important by African-Americans and least likely to be rated as important by Asian-Americans after adjusting for sex (all with *p* < 0.05).

Item (7)(a) “donating blood is painful” was the most commonly chosen item of influential deterrence (13%), whereas item (7)(d) “donating blood takes too much time” was the least commonly chosen potential deterrent (6%). Item (7)(d) was more likely to be rated as influential by males than by females after adjusting for race. Item (7)(c) (dislike the sight of blood) was most likely to be rated as influential by African-Americans, followed by Hispanics or Caucasians, and lastly by Asians after adjusting for sex. Item (7)(g) “The nurses/staff were not friendly” was most likely to be rated as influential by African-Americans and least likely to be related as influential by Asians after adjusting for sex (all with *p* < 0.05).

Table 2 lists the unadjusted and the corresponding adjusted percentages by race, sex, and overall for question #(8) which asked respondents to rate the attractiveness of various incentives.

Items (8)(a) “movie tickets” (83%) and (8)(b) “free cookies/snacks” (83%) were rated appealing most likely, whereas item (8)(f) “chance to erase some of my tardies to class” was least likely to be rated as appealing. Aside from males being more likely to rate this as appealing compared to females, there were no other notable differences seen due to sex or race in influencing the responses to question #(8).

In general, there were no notable interactions between sex and race in influencing the responses to any of the questions.

## 4. Discussion

Maintaining an adequate blood supply to support the aging, ethnically diverse US population depends on being able to successfully recruit individuals to donate blood. Understanding the variety of individual beliefs about different aspects of blood donation and the priority that those beliefs play in the decision to donate is essential to designing effective recruitment strategies.

Our study surveyed student blood donors at four local high school blood drives. The high schools chosen were geographically separate and socioeconomically variable. The demographic data collected demonstrated diversity among survey respondents. Male and female sexes participated equally, and racial diversity was reflected by over 75% of respondents identified as a minority (African-American, Hispanic/Latino, and Asian-American). The fastest growing ethnic minorities in the US [7] were well represented among our study group with over half (66%) of all respondents identifying themselves as either Asian-American or Hispanic/Latino. Finally, respondents were diverse in their blood donation experience with slightly over half reporting being first-time donors and almost a fourth having donated twice or more.

Consistent with numerous reports of blood donor motivation [10–12, 14–17, 19], overall, motives that reflect prosocial beliefs were rated as the most important motivators in the respondents' decision to participate in the blood drive. Prosocial beliefs may be altruistic in nature (helpfulness) or empathetic (feeling for others) or may be considered part of social responsibility (a duty to help others) [11]. Respondents rated items that reflect these concepts highly. Other highly rated motivators were feeling like a hero, someone being proud of them, the fact that their friends were participating, and helping their school collect more blood than other schools.

Previously, it has been argued that the Millennial generation (people born between 1981 and 2000, as were the respondents of this survey) has lesser civic engagement than the previous generations; it has been speculated that this decline in “social capital” may be associated with a loss of altruism in this age group, which is encouraged by community involvement [20]. Thus, it might have been speculated that items highlighting the personal gain of the blood donation process (such as a receiving a gift or the opportunity to socialize with friends) may have been comparatively more motivating to this younger age group; this, however, was not found. The social aspect of the drive (item (5)(k)) was chosen as a motivating factor by only 40.8% of the respondents overall, and the gift/incentive was chosen as an influence by the least number of participants (30%) overall. This is in contrast to past reports of college aged blood donors, in which the gift/incentive was the third most highly chosen motivator and was more important to younger aged donors compared to older donors [17].

The concept of donating blood to help one's school collect more than other schools was chosen as an important motivator by almost half of all respondents. This item represents blood donation as an opportunity for competition and achievement on an institutional level and may uniquely inform the development of a recruitment strategy targeted to this age group. While there are other instances of donations being made in group settings to achieve a certain number of collections on an institutional level (e.g., a mobile blood drive at a business office in which information regarding the total collections from that drive is disseminated to the individuals that work there as a matter of organizational pride), the concept of one's organization being in direct competition with similar local organizations is somewhat unique to high schools, possibly engendered by interschool activities such as sports programs, in which “school spirit” is an important element. The competitive aspect of this activity may be explored in recruitment campaigns that highlight and praise specific schools that donate the most units of blood in a particular school district.

In regard to motives, some differences between demographic groups were detected. As far as differences between the sexes go, more male than female respondents responded positively to the items “donating blood is the right thing to do” and “my friends are donating,” which demonstrates the importance of both of these factors to male donors in this age group. In past studies of youthful (college aged) blood donors, while altruism was the most important motivator of donation behavior regardless of sex or race, women were reported to be more likely to be motivated by the idea of helping others and to report being influenced by family and friends, whereas men appeared to be more influenced by social pressure and did not want to disappoint others [12, 17]. In our survey, males and females were equally likely to report “I'd feel bad if I did not” as important in their decision to donate.

Some motivating factors were particularly important to African-Americans compared to other races, including “donating blood makes me feel like a hero” (also rated positively significantly by Hispanic/Latino respondents), “other kids in my club/sport are donating,” “adults at my church/community/school donate,” and “I want to help my school donate more than any other school.” These items reflect donating as an empowering experience that is performed by individual members of larger organizations. It is possible that these concepts are more familiar to individuals of a minority ethnicity or may reflect cultural differences.

The pain associated with donation was the most commonly rated potential deterrent. Other reports of college aged blood donors have also reported this as a deterring factor of influence [12, 17]. The concept of phlebotomy may cause anxiety or fear in a younger age group because, compared to older blood donors, they have overall less experience with medical intervention and the associated venipuncture procedure. Staff and nurses collecting blood at high school drives should be aware of this and aim to manage and assuage needle-associated anxiety.

Inconvenience and time constraints, which have been found to be significant deterrents in college aged [12, 17] and older donors [16], were the deterrents least commonly chosen as influential by this age group. It seems likely that the presence of the drive at a location where the students are already required to be diminishes the inconvenience of this experience.

In regard to potential deterrents, some differences were found between sexes and races. Males were more likely than females to rate the time involved in blood donation as influential as a deterrent. African-Americans were more likely to be deterred by the sight of blood or the unfriendliness of the blood drive staff. Past studies examining racial differences in beliefs about blood donation have reported that African-Americans are more likely to report agreement with statements that reflect distrustfulness of medical establishments and that this concern affects the willingness to donate blood [15, 21]. Thus, recruitment strategies and staff behavior that focus on engendering a trusting relationship between potential blood donors and the collection staff and nurses may be effective in avoiding deterrence of young African-American donors.

Regarding potential incentives given in gratitude of blood donation, concrete items were more frequently rated as appealing compared to nonmaterial rewards.

Consistent with past reports of younger age groups [17], movie tickets were one of the most common favorably rated gifts. Movie tickets remain a gift of high appeal likely because they are somewhat valuable and are easily redeemable.

Free cookies and snacks (which were given both prior to and after donating) were rated appealing as frequently as movie tickets. Although not substantiated, edible gifts are generally considered to play a role in supporting the circulating glucose concentration of the donor after the donation experience; they have not been posed as potential incentives to donate in past studies of older donors. It is thus not known if this form of incentive would be similarly popular among other age groups, and it is difficult to speculate why they might have been so favorably rated by the respondents in this survey. This could represent a tendency for a younger age group to choose a gift that imparts instant gratification, although this is speculative.

Although the nonmaterial rewards proposed by the survey represented creative forms of incentive, some of which were specifically targeted to a high school student's interests, they were still less popular than concrete rewards. Offerings such as hours towards community service requirements and a chance to erase one's tardies to class were rated as appealing least frequently. Free health screenings, which have been reported as popular among older donors, particularly Black and Hispanic donors [10], were less popular among this age group, regardless of race. These findings suggest that recruitment strategies aimed at high school aged donors may be more successful if they focus on the instant, concrete gifts given in appreciation of one's blood donation, rather than on nonmaterial rewards.

This study has some limitations. First, the number of respondents of some races (African-American and Asian) was low compared to the total; the relatively low amount of data collected from these groups may have caused a failure to detect significant differences of these groups compared to the others.

Secondly, as it was conducted at one discrete time point in the blood donation process, it only examines the donors' experiences at this particular time (right after donation). For the motivating factors, it relies on donors to remember their initial intention to donate blood; the experience of donating may have changed their perceptions. Additionally, donors may experience delayed reactions or discomfort after the blood drive which may deter them from future donations, and this survey, administered in the canteen area, would not capture this.

Third, the survey was somewhat limited in content; this was by design, as we felt that a survey that was too lengthy would not be as successful in achieving an adequate rate of complete responses. Also, as it was conducted in the canteen area, the survey had to be completed by a respondent in less than 15 minutes; otherwise, it would have created a backflow affecting the influx of donors to the area.

Finally, as this survey was administered only to students who had just donated, there was no opportunity to examine the motives or deterrents of those that did not choose to participate in the blood drive.

This study is significant in that it is the first study to examine multiple potential motivating and deterring factors in this particular age group. Another strength of this study is the ethnic diversity of its respondents. The findings of this study may be used to design recruitment campaigns to inspire young blood donors.

Highlighting the altruistic aspects of blood donation, as well as the community need for blood, and the social responsibilities fulfilled by donating blood could be effective in recruiting and maintaining an ethnically diverse group of blood donors of this age group.

## Figures and Tables

**Table 1:** Potential motivating factors (A) and deterrents (B) for blood donation.

	Q	Comparisons by sex			Comparisons by race					
		Adjusted, %^**∗**^			Adjusted, %					
		Female	Male	*p* value^‡^	Afr-amer	Asian	Cauc	Hisp	Other	*p* value^§^
		*n* = 206	*n* = 189		*n* = 36	*n* = 76	*n* = 71	*n* = 186	*n* = 26	
*(A) Potential motivating factor*										
Donating blood is the right thing to do	(5)(a)	85.2	94.2	0.026	90.9	92.5	92.9	89.2	86.3	0.841
Donating blood makes me feel like a hero	(5)(b)	58.5	64.0	0.403	66.9	51.3	61.8	71.7	53.6	0.025
There is a shortage of blood for people that need it	(5)(c)	75.4	75.2	0.973	82.4	75.2	74.6	68.1	75.0	0.450
Donating blood is good for my health	(5)(d)	29.8	39.4	0.136	28.3	34.2	39.7	34.8	35.5	0.883
My friends are donating	(5)(e)	45.7	66.1	0.004	73.6	50.1	45.8	49.3	60.0	0.130
Other kids in my sport/club are donating	(5)(f)	35.2	48.3	0.059	67.7	25.0	47.4	34.7	35.4	0.001
Adults at my church/school/community donate blood	(5)(g)	35.5	39.0	0.600	60.4	27.6	38.1	31.0	31.3	0.038
Someone in my family is a blood donor	(5)(h)	39.6	36.7	0.668	56.9	24.4	38.1	37.2	36.4	0.050
Someone will be proud of me if I donate	(5)(i)	52.7	64.7	0.072	66.9	44.7	63.3	61.5	57.1	0.093
I want to help my school donate more than any other school	(5)(j)	47.0	50.8	0.589	65.9	39.1	43.9	55.6	39.8	0.033
I like hanging out with my friends at the blood drive	(5)(k)	41.4	46.0	0.491	55.1	30.3	46.9	43.4	—	0.074
I wanted the gift/incentive	(5)(l)^†^	30.5	36.1	0.364	37.8	30.1	31.1	28.4	39.8	0.729
I'd feel bad if I didn't	(5)(m)	53.1	45.0	0.229	54.7	46.0	49.2	43.3	51.9	0.761

*(B) Potential deterrent*										
Donating blood is painful	(7)(a)	13.5	11.1	0.556	14.6	10.5	17.0	12.6	6.7	0.364
Donating blood is inconvenient	(7)(b)^†^	5.6	8.2	0.405	3.8	7.9	11.5	7.8	3.3	0.483
I don't like the sight of blood	(7)(c)	12.9	7.9	0.233	27.7	4.0	11.1	9.2	0.0	<0.001
Donating blood takes too much time	(7)(d)	2.3	7.8	0.039	3.8	6.8	3.9	6.2	4.5	0.856
I don't like missing class to donate blood	(7)(e)	7.6	8.0	0.906	12.7	6.5	8.1	7.3	4.5	0.831
I felt badly after I donated blood (light headed, dizzy, etc.)	(7)(f)	9.4	8.6	0.809	5.8	10.5	14.0	8.1	6.7	0.457
The nurses/staff were not friendly	(7)(g)	7.0	10.7	0.301	20.4	1.3	3.9	13.9	4.5	<0.001

^*∗*^Percentages shown are the combined percentages of respondents who indicated very important/somewhat important for question (5) or very influential/somewhat influential for question (7).

^†^Questions (5)(l) and (7)(b) each have 1 missing response.

^‡^The *p* values in this column correspond to the main effect of sex under the adjusted model.

^§^The *p* values in this column correspond to the main effect of race under the adjusted model.

**Table 2:** Perceived attractiveness of various incentives.

	Q	Comparison by sex				Comparison by race					
		Adjusted, %^**∗**^				Adjusted, %					
		Overall	Female	Male	*p* value^‡^	Afr-Amer	Asian	Cauc	Hisp	Other	*p* value^§^
		*n* = 395	*n* = 206	*n* = 189		*n* = 36	*n* = 76	*n* = 71	*n* = 186	*n* = 26	
*Type of incentive*											
Movie tickets	(8)(a)	82.5	87.9	82.5	0.292	89.3	90.7	80.2	77.9	—	0.102
Free cookies/snacks after donation	(8)(b)^†^	83.0	88.2	84.7	0.475	89.3	92.1	78.6	79.4	88.8	0.094
Logo t-shirts, towels, mugs, bags	(8)(c)	75.7	81.8	73.6	0.149	82.4	84.9	70.3	72.4	77.1	0.197
Free health screening (i.e., cholesterol level, diabetes test)	(8)(d)	76.5	79.5	74.1	0.371	83.2	84.2	72.8	76.3	64.5	0.245
Credit towards community service hours performed	(8)(e)	74.9	73.0	81.6	0.147	71.5	87.1	73.5	70.6	81.7	0.093
A chance to erase some of my tardies to class	(8)(f)	59.0	51.1	69.7	0.006	66.9	53.1	51.7	61.9	69.4	0.309

^*∗*^Percentages shown are the combined percentages of respondents who indicated appealing/somewhat appealing.

^†^Question (8)(b) has one missing response.

^‡^The *p* values in this column correspond to the main effect of sex under the adjusted model.

^§^The *p* values in this column correspond to the main effect of race under the adjusted model.

## Appendix

### Survey Instrument

*Section  1. Demographic Data*

1. What is your sex?
  Male
  Female
2. What is your age?
  16
  17
  18
  19
3. What is your race?
  African-American/Black
  Asian/Pacific Islander
  Caucasian/White
  Hispanic/Latino
  Mixed or Other, please specify:
4. How many times have you donated before?
  None, this is my first time
  Once before
  2-3 times before
  More than 3 times before

*Section  2. Motives and Deterrents to Donation*

(5) Of the list of factors below, which of the following were important in your decision to donate blood today? ((1) Very unimportant, (2) Somewhat unimportant, (3) Neutral/not sure, (4) Somewhat important, (5) Very important)
  - Donating blood is the right thing to do.
  - Donating blood makes me feel like a hero.
  - There is a shortage of blood for people that need it.
  - Donating blood is good for my health.
  - My friends are donating.
  - Other kids in my club/sport are donating.
  - Adults at my school/church/community donate blood.
  - Someone in my family is a blood donor.
  - Someone will be proud of me if I donate.
  - I want to help my school to donate more than any other school.
  - I like hanging out with my friends at the blood drive.
  - I wanted the gift/incentive.
  - I'd feel bad if I did not.
(6) How likely are you to donate blood again?
  Very likely
  Somewhat likely
  Not sure
  Probably not
  Definitely not
(7) Which factors below would most likely influence you to not donate again? ((1) No influence, (2) Not much influence, (3) Neutral/not sure, (4) Somewhat influential, (5) Very influential)
  - Donating blood is painful.
  - Donating blood is inconvenient.
  - I do not like the sight of blood.
  - Donating blood takes too much time.
  - I do not like missing class to donate blood.
  - I felt badly after I donated blood (light-headed, nauseated, dizzy, faint, etc.).
  - The nurses/staff were not friendly.

*Section  3. Appeal of Gifts/Incentives*

(8) Should you donate blood again, which incentives would you find most appealing? ((1) Appealing, (2) Somewhat appealing, (3) Neutral/not sure, (4) Not appealing)
  - Movie tickets.
  - Free cookies/snacks after donation.
  - Items with the UCLA Blood and Platelet Center logo such as T-shirts, towels, mugs, or bags.
  - Free health screening, such as for cholesterol level or a diabetes test.
  - Credit towards community service hours I've performed.
  - A chance to erase some of my tardies to class.
